# Impact of Limited Sample Size and Follow-up on Partitioned Survival and Multistate Modeling-Based Health Economic Models: A Simulation Study

**DOI:** 10.1177/0272989X251342596

**Published:** 2025-06-25

**Authors:** Jaclyn M. Beca, Kelvin K. W. Chan, David M. J. Naimark, Petros Pechlivanoglou

**Affiliations:** Institute of Health Policy, Management and Evaluation, University of Toronto, Toronto, Canada; Canadian Centre for Applied Research in Cancer Control (ARCC), Toronto, Canada; MORSE Consulting Inc, Toronto, Canada; Institute of Health Policy, Management and Evaluation, University of Toronto, Toronto, Canada; Canadian Centre for Applied Research in Cancer Control (ARCC), Toronto, Canada; Sunnybrook Health Sciences Centre, Toronto, Canada; Institute of Health Policy, Management and Evaluation, University of Toronto, Toronto, Canada; Sunnybrook Health Sciences Centre, Toronto, Canada; Institute of Health Policy, Management and Evaluation, University of Toronto, Toronto, Canada; Child Health and Evaluative Sciences, Hospital for Sick Children, Toronto, Canada

**Keywords:** Decision models, economic evaluation, partitioned survival models, multistate models, survival analysis, simulation

## Abstract

**Background:**

Economic models often require extrapolation of clinical time-to-event data for multiple events. Two modeling approaches in oncology that incorporate time dependency include partitioned survival models (PSM) and semi-Markov decision models estimated using multistate modeling (MSM). The objective of this simulation study was to assess the performance of PSM and MSM across datasets with varying sample size and degrees of censoring.

**Methods:**

We generated disease trajectories of progression and death for multiple hypothetical populations with advanced cancers. These populations served as the sampling pool for simulated trial cohorts with multiple sample sizes and various levels of follow-up. We estimated MSM and PSM by fitting survival models to these simulated datasets with different approaches to incorporating general population mortality (GPM) and selected best-fitting models using statistical criteria. Mean survival was compared with “true” population values to assess error.

**Results:**

With near complete follow-up, both PSMs and MSMs accurately estimated mean population survival, while smaller samples and shorter follow-up times were associated with a larger error across approaches and clinical scenarios, especially for more distant clinical endpoints. MSMs were slightly more often not estimable when informed by studies with small sample sizes or short follow-up, due to low numbers at risk for the downstream transition. However, when estimable, the MSM models more commonly produced a smaller error in mean survival than the PSMs did.

**Conclusions:**

Caution should be taken with all modeling approaches when the underlying data are very limited, particularly PSMs, due to the large errors produced. When estimable and for selections based on statistical criteria, MSMs performed similar to or better than PSMs in estimating mean survival with limited data.

**Highlights:**

- Caution should be taken with all modeling approaches when underlying data are very limited.
- Partitioned survival models (PSMs) can lead to significant errors, particularly with limited follow-up. Incorporating general population mortality (GPM) via internal additive hazards improved estimates of mean survival, but the effects were modest.
- When estimable, decision models based on multistate modeling (MSM) produced similar or smaller error in mean survival compared with PSM, but small samples or limited deaths after progression produce additional challenges for fitting MSMs; more research is needed to improve estimation of MSMs and similar state transition–based modeling methods with limited data.
- Future studies are needed to assess the applicability of these findings to comparative analyses estimating incremental survival benefits.

Economic evaluations are routinely used in health technology assessment (HTA) to examine the cost-effectiveness of technologies compared with relevant alternatives. Economic evaluation decision models estimate the expected benefits and costs of technologies often over the lifetime of patients with chronic diseases such as cancer. Parameter inputs are typically informed by time-to-event endpoints from clinical trials. However, such trials almost always have follow-up times that are significantly shorter than the time horizons used in the decision models. Therefore, extrapolation from the observed trial data is necessary, which is commonly achieved using parametric survival analysis. Given the complexity of interventions and their effects, particularly in oncology, it is often valuable to incorporate time dependency in such extrapolations. Decision models, especially in oncology, include intermediate and terminal health events (e.g., progression and death); therefore, multiple such parametric survival analyses are needed. Transitions to health states can also be competing, which correspondingly requires the use of competing risk survival analysis methods.

Time dependency can be incorporated into decision models using semi-Markov assumptions. The parameters of such decision models can be estimated using multistate frameworks.^1^ In particular, parametric distributions can be fit to observed event-time data to estimate hazards for each event. In medical oncology, a common multistate scenario involves patients starting with limited disease (being in a progression-free [PF] state), progressing to a state of increased cancer burden (being in a progressed disease [PD] state), and then dying (entering the death state). A multistate model estimates continuous-time hazards for each possible transition between a set of finite health states or events.^1,2^ Multistate survival parameters can be coupled with individual-level simulation models to estimate disease processes comprehensively within a common framework. Simulation methods such as discrete event simulation (DES) can be used to extrapolate outcomes and estimate mean survival.^3,4^ In keeping with other examples in the related literature, we refer to the semi-Markov decision model coupled with multistate framework for parameter estimation as the multistate modeling (MSM) approach.^4–6^

In economic models informing HTA in medical oncology, a more commonly used alternative model structure is a partitioned survival model (PSM).^7^ In PSM, parametric survival models are fitted to overall survival (OS) and progression-free survival (PFS) data, and estimates of state occupancy for preprogression, postprogression, and death are estimated using survival probability for both outcomes throughout the PSM’s time horizon. In other words, extrapolated PFS and OS survival curves are used to decompose the probability of occupying each health state and integrated to estimate mean survival.^7^ This method has often been favored due to its ability to capture time dependency reflected in observed trial PFS and OS data. The main challenge for the PSM framework is its lack of the theoretical underpinnings in decision modeling to plausibly capture expected risks for relevant health state transitions in the extrapolated period.^7^

The structural differences and the associated assumptions of these 2 types of approaches (PSM and MSM) have been considered in application settings, and properties have been compared but outcomes have not been systematically examined through a simulation study.^5,7–9^ We hypothesize that the ability of each model type to adequately capture the true survival is dependent on the quality and quantity of underlying data and analysis choices, particularly given the need to extrapolate to a lifetime horizon.^10,11^ Hazards from within a trial with incomplete follow-up may not be representative of long-term hazards and can lead to erroneous or implausible outcomes.^10–13^ Previous research has demonstrated that estimation of the long-term hazards when extrapolating is improved by incorporating general population mortality (GPM) hazards in the log-likelihood function of standard parametric distribution.^14^

The overall aim of this study was to assess the performance of PSM- and MSM-based approaches when extrapolating incomplete data of various sizes and degrees of censoring. To achieve this, we 1) generated cancer disease trajectories for a population of trial participants with known, true sojourn times in 3 states (PF, PD, dead); 2) randomly selected individuals from the population to enroll in simulated trials of various size and degrees of censoring; 3) employed each trial’s data to derive PSM and MSM simulation models, including the impact of incorporating GPM; and 4) assessed the performance of PSM and MSM approaches.

## Methods

### Simulation Setup

#### Model structure for data generation

**Figure 1:**
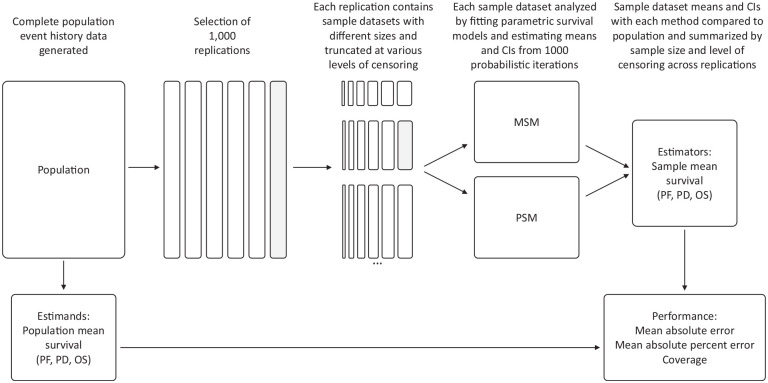
Overview of simulation study design.

We designed a simulation study generating data for events of progression and death to mimic the outcomes of a population with a diagnosis of late-stage cancer (Figure 1, Table 1). Population data were first generated from a 5-state multistate process.^15,16^ We simulated from a progressive disease model with health states for PF, PD, and death, with no backward transitions (See Appendix Figure 1 for more details).^7^ A transition between pre-trial and PF health states was used to generate random staggered enrollment times into the trial. We represented death with 2 states to account for hazards for death based on an estimated background GPM as well as excess disease-specific mortality (DSM). We assume that for an individual with the disease, the overall mortality risk is determined from the sum of background GPM and excess DSM hazards.

**Table 1.** Simulation plan according to ADEMP guidelines

Category	Description
Aims	Assess the performance of partitioned survival models (PSM) and Semi-Markov decision-models estimated using multistate models (MSM) when extrapolating incomplete data of various sizes and degrees of censoring.
Data generating mechanism	Data were generated for populations with 50,000 patients for each scenario from a set of survival distributions in a multistate process. See Table 2.
Population estimands	Mean population time in each state (PF, PD) and total survival time (OS = PF + PD)
Methods	Each of $n_{\mathrm{sim}}=1,000$ replications included 1,050 patients allocated into cohorts of six sample size groups ( $n_{\mathrm{obs}}=\{30,\;60,\;90,\;120,\;250,\;500\})$ . Each cohort was repeatedly artificially truncated to form eight levels of follow-up completeness based on proportion of events observed, of $\left(p_{e}\;=\;\left\{30\%,\;40\%,\;\ldots 100\%\right\}\right)$ . Each replication was analyzed with each level of sample size and proportion of events observed, resulting in 6 sample sizes x 8 levels of completeness x 1,000 replications = 48,000 sample datasets analyzed for each scenario. Standard parametric distributions (Weibull, log-normal, log-logistic, generalized gamma and Gompertz) were fitted in each dataset: • For PSM, to PFS and OS data. For OS, the hazard was either fitted to observed data alone (naïve PSM), or relative to general population mortality (GPM) (PSM with GPM). • For MSM, for events of PD, death from PF, death from PD. Both hazards for death were estimated for the disease and fitted relative to GPM hazards for death from other causes (MSM with GPM). The model was also estimated in a simplified manner by assuming only GPM hazards for death from PF (Partly naïve MSM). Nonconverging fits for specific distributions were removed and best-fitting curves were chosen by AIC or BIC (corrected for small samples) for each event/transition. For MSMs, sojourn times were estimates for 10,000 individuals with DES to capture first-order uncertainty. Mean sojourn times spent in PF, in PD, and total survival time (PF+PD) and their 95% CIs were estimated with second order uncertainty based on 1,000 probabilistic iterations drawn from multivariate normal distributions of the best-fitting survival parametric distribution parameters from PSMs and MSMs for each sample dataset. The means and CIs from each sample dataset were summarized by sample size and proportion of event groupings across the 1000 replications and compared to the true population estimate to estimate performance.
Performance measures	Error: Mean absolute percent error, mean absolute error, bias Coverage

#### Model parameters for the simulated data-generation process

Randomly generated enrollment times $t_{1i}$ were sampled from a uniform distribution to a maximum accrual time, $T_{1}$ (1.5 y). Event times for individuals were generated in years. The population hazard for death from PF was assumed to be represented by historical data on GPM, $\lambda_{\mathrm{pop}\_\mathrm{gpm}}\left(t\right)$ . The hazard $\lambda_{\mathrm{pop}\_\mathrm{gpm}}\left(t\right)$ was generated from life tables from Statistics Canada fitted to a historical year (1980/82) using a Kannisto–Makeham mortality law.^17^ The reason for this choice of data and distribution was 2-fold; first, we selected GPM data to capture a realistic hazard for death without disease progression but intentionally introduced a difference between the hazard used to generate the population and the GPM hazards used in analysis of samples (approach to GPM hazards used in analysis described below, intentionally different using a Gompertz distribution with more recent 2017/19 data). Second, given lower life expectancy several decades ago, the population hazard of death from other causes is higher than contemporary GPM and thus a potentially more plausible total hazard estimate for patients with an advanced disease (Appendix Figure 2).

The total hazards for transitioning from PD to death was represented by the sum of population GPM hazards $\lambda_{\mathrm{pop}\_\mathrm{gpm}}(t)$ and excess DSM hazards $\lambda_{\mathrm{pop}\_\mathrm{dsm}}\left(t\right).$ Each individual in the cohort had an estimated baseline age that was randomly generated from a normal distribution with a mean of 60 y (standard deviation = 6) to estimate their individual baseline $\lambda_{\mathrm{pop}\_\mathrm{gpm}}(t)$ . Hazards for the transition from PF to PD and excess mortality from PD to death from disease were generated using exponential distributions in scenario 1; we varied the hazards in additional scenarios to explore different clinical risk patterns, with increasing risks over time for progression and excess DSM mortality from PD (scenario 2), decreasing risk of progression (scenario 3), and decreasing risk of progression but overall lower absolute risks compared with scenario 3 (scenario 4) (Table 2).

**Table 2.** Description and data generating parameters used for the four population scenarios

		$\lambda_{\mathrm{PF}\to \mathrm{PD}}(t)$		$\lambda_{\mathrm{dsm}}(t)$		$\lambda_{\mathrm{gpm}}(t)$
	Scenario description	Rate (scale)	Shape	Rate (scale)	Shape	
1	Constant (exponential) for both progression and excess risk of death	0.5	1.0	0.2	1.0	Kannisto-Makeham mortality law using Statistics Canada life tables, (1980/82).[17] Baseline ages from normal distribution, mean 60 years (standard deviation = 6)
2	Monotonically increasing (Weibull) for both progression and excess risk of death	0.5	**1.6**	0.2	**1.6**	
3	Monotonically decreasing (Weibull) for progression, monotonically increasing (Weibull) for excess risk of death	0.5	**0.4**	0.2	1.6	
4	Monotonically decreasing (Weibull) for progression, monotonically increasing (Weibull) for excess risk of death, scenario 3 with lower rates	**0.1**	0.4	**0.1**	1.6	

gpm = general population mortality; dsm = disease-specific added mortality

#### Simulated populations

We simulated event history data for 50,000 individuals from the described data-generating process to form our complete populations for each of the 4 scenarios described above. The event history data included the events of enrollment (start time in PF), progression, and death. We used time of enrollment to reset clocks to zero to generate staggered follow-up and censoring times.

#### Simulated samples

We randomly selected $\mathrm{sum}(n_{\mathrm{obs}})=1,050$ patients from each population, and allocated them into 6 sample size groups, $n_{\mathrm{obs}}=\left\{30,\;60,\;90,\;120,250,\;500\right\}$ . Each random draw represented 1 replication, and 1,000 replications were sampled with replacement to simulate random selections of patients from the population entering clinical trials.

To assess varying levels of follow-up completeness across scenarios and replications, we determined times at which various proportions of the total sample had experienced an event in each sample. We focused on the proportion of PFS events (first event of progression or death) observed, to inform a scenario in which the PFS is the primary outcome of a clinical trial and number of PFS events is used to determine the timing of data cutoff for analysis. Based on previous studies in the literature, we examined follow-up completeness after at least 30% of the cohort had experienced PFS to enable extrapolation and ensure some observations for OS.^10^ Within each replication and sample size, we created datasets that were censored at study times associated with different proportions of events, $p_{e}=\{30\%,40\%,\ldots ,\;100\%\}$ , creating 8 levels of follow-up completeness. We refer to each combination of sample size and level of events observed as a “grouping.”

Thus, each scenario included $n_{\mathrm{sim}}$ = 1,000 replications. Within each replication, there were 6 levels of $n_{\mathrm{obs}}$ , which in turn were evaluated with 8 levels of $p_{e}$ , producing a total of 48,000 “sample datasets” for analysis.

### Analysis Methods

#### Estimands

The complete population data were used to generate mean survival time estimands for the population. We fit nonparametric Kaplan–Meier curves to the PFS and OS data. The time at which the population’s observed OS indicated that almost all individuals have experienced the event (set at 1%) was used to define the maximum follow-up time horizon for estimating the mean survival time for each scenario, similar to previous studies.^10^ The mean OS and the time spent in PF or PD states for the population was determined from the area under the complete population Kaplan–Meier curves to the established time horizon.^18^

#### Estimators

The mean survival time was estimated for each grouping across replications using 2 model frameworks, PSM and MSM. The data in each sample were fitted with 6 standard parametric distributions: exponential, Weibull, log-normal, log-logistic, generalized gamma, and Gompertz. The best-fitting distributions in each case were selected based on lowest Akaike information criterion corrected for small sample sizes.^19^ The specific events for each model type are described below.

##### PSM

In the PSM, the time-to-event outcomes included PFS and OS. PFS was defined as the time from entering the study to the first event of progression or death; OS was defined as the time from entering the study to reaching the death state, regardless of trajectory.

The hazard for progression or death from PF was estimated from observed data with the 6 standard parametric distributions. The OS hazards were estimated using a combination of baseline GPM hazard and the relative excess DSM hazard.^20–22^ The baseline GPM hazard was estimated from a Gompertz model fitted to Canadian life table data from a starting age of 60 y.^20,21^ The excess DSM hazard was estimated relative to GPM with the 6 standard parametric distributions.^22^ We acknowledge that age-related GPM includes disease-specific causes, but the contribution of a specific cancer to the GPM estimates is expected to be very small. This is often considered appropriate in practice; however, if not appropriate in a certain disease context, modifications to the GPM would be necessary.^23^ Thus, the total hazard for death from PD was defined as $h\left(t\right)=h^{*}\left(t\right)+\;\lambda (t)$ , where $h^{*}\left(t\right)$ was the baseline GPM hazard function and λ(t) was the excess DSM hazard function estimated with a parametric distribution, and thus, the OS survival function was expressed as $S\left(t\right)=S^{*}\left(t\right)*R(t)$ , where $S^{*}\left(t\right)$ was the baseline survival from GPM hazards and R(t) was the relative survival estimated with a parametric distribution.^22^ This approach is most commonly known as internal additive hazards.^14^

We compared the results of incorporating baseline GPM hazards in this manner with no GPM adjustment, wherein OS was estimated based on the hazards derived from the parametric distribution fitted to the simulated trial data. We refer to the former as “PSM with GPM” and the latter as “naïve PSM” without baseline hazard, as it is the historically typical approach used.^7^

Given that the 2 trial outcomes that PSM relies on are correlated (OS and PFS) but also that conventional parametric survival estimation treats them as independent, implausible extrapolations of survival for these outcomes can occur. Most commonly, PFS can be estimated to be greater than OS in the extrapolated portion of the PSM. In such circumstances, the PSM will produce implausible estimates of PD (Pr(PD) < 0). For that purpose, we followed a common approach in practice in which the PSM was corrected such that if PFS≤OS,PFS=PFS,andifPFS>OS,PFS=OS at every cycle, if required.^24^ The area under the PFS and OS survival functions were integrated at monthly cycle intervals to estimate survival in each state; the area under the OS curve represented total survival or life-years, the area under the PFS curve represented time spent in PF, and the difference represented time spent in PD. The mean survival and 95% confidence intervals were estimated with second-order uncertainty captured using 1,000 probabilistic iterations drawn from multivariate normal distributions of the best-fitting parametric distribution parameters.

##### Semi-Markov decision model using multistate model parameters

The MSM structure included events of progression, death from other causes, and death from disease to conceptualize the disease process and capture GPM using a 5-transition matrix (see Appendix Figure 1). We estimated hazards for death from PF and PD using a combination of baseline GPM and relative excess DSM hazards.^20–22^ Relative excess hazards were estimated using the best-fitting of 6 standard parametric distributions fitted to the at-risk dataset for each transition. Where there was no estimable relative survival model to capture excess DSM, we used a 4-transition matrix without the relative excess DSM hazards to death from PF (reducing the 3 possible hazards from PF to simply progression or GPM hazards to death). We refer to this approach as “MSM with GPM.”

We compared the results of fully capturing GPM in the transition matrix with a simplified model using a 4-transition matrix, simply using the observed hazards (ignoring GPM) from PF to death while still estimating both GPM hazards and relative excess DSM hazards to death from PD. We refer to this simplified approach as “partly naïve MSM.” For both approaches, a grouping’s replication was discarded if all 6 parametric distributions failed to converge for 1 or more hazard estimations.

This model includes data analysis using the MSM framework and individual-level simulation that corresponds to a DES according to a “first event to occur” approach using the cause-specific hazards framework. Continuous-time individual DES using *flexsurv* in R was used to simulate the sojourn time in specific states from the set of fitted survival models.^22^ Event times for each possible event were randomly generated, and the first of competing events to occur was retained to determine the event trajectory and time for each individual. We captured stochastic and parameter uncertainty with 2 levels of simulation: 1) first-order uncertainty was captured using the best-fitting survival model parameters for each event hazard, and event times for 10,000 simulated individuals were generated to estimate average sojourn times for 1 iteration and 2) second-order uncertainty was captured using 1,000 probabilistic iterations drawn from multivariate normal distributions of the survival model parameters to generate mean estimates and 95% confidence intervals for PF, PD, and OS survival time.

### Analysis

The mean survival time from each grouping and replication were compared with the full population mean survival estimands for OS, PF, and PD to estimate the mean absolute percentage error (MAPE), mean absolute error, and coverage across all the replications in the simulation.

R statistical software (v 4.0.4) was used to simulate and analyze data, using the *gems* package to generate the data, *flexsurv* and *survHE* to fit parametric survival models, *darthtools* for PSM, and *flexsurv* to fit parametric MSM hazards and estimate length of stay in each health state with DES.^22,24–26^

### Role of Funding Source

No funding was received for this study.

## Results

### Populations

Four disease scenarios with varying event hazards for progression and death prior to and following progression produced a range of mean survival time estimands (Table 3, see Appendix Figures 3 and 4 for population survival curves and hazards for death with or without baseline hazards incorporated).

**Table 3.** Estimands for mean population survival for each scenario in years and time horizon used (maximum time used to estimate mean survival in the population and across all replications for the scenario).

	1 Constant risk of progression and death	2 Increasing risk of progression and death	3 Decreasing risk of progression, increasing risk of death	4 Similar to Scenario 3 with lower baseline risks
PF	1.95	1.77	3.69	7.91
PD	4.38	4.16	3.80	5.57
OS	6.33	5.92	7.49	13.48
Time horizon	22.0	14.0	32.0	41.0

### Model Estimation

**Figure 2:**
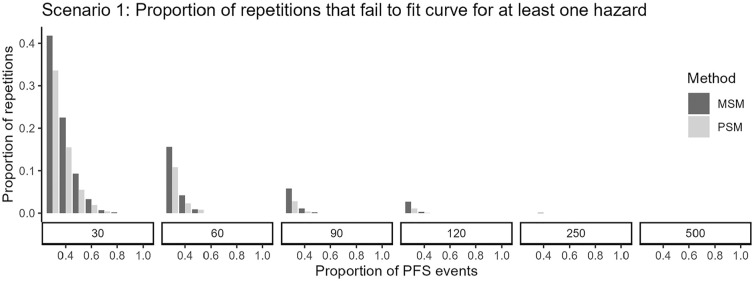
Proportion of replications failing to converge (i.e., where for at least 1 of the hazards to be estimated, none of the 6 distributions successfully fit) in scenario 1 for PSM and MSM approaches. MSM, multistate model; PSM, partitioned survival model.

We observed that when fitting parametric distributions to datasets with small sample sizes, the estimation of at least 1 event hazard failed somewhat more often for MSM approaches, particularly when follow-up completeness was limited (Figure 2). Patterns were similar across scenarios, but there was a larger number of repetitions that failed in scenarios 3 and 4 with small samples and a larger discrepancy in the amount of failures between MSM and PSM approaches in scenario 2 and in scenario 4 with limited follow-up (Appendix Figure 5).

### Extrapolated Survival Performance

**Figure 3:**
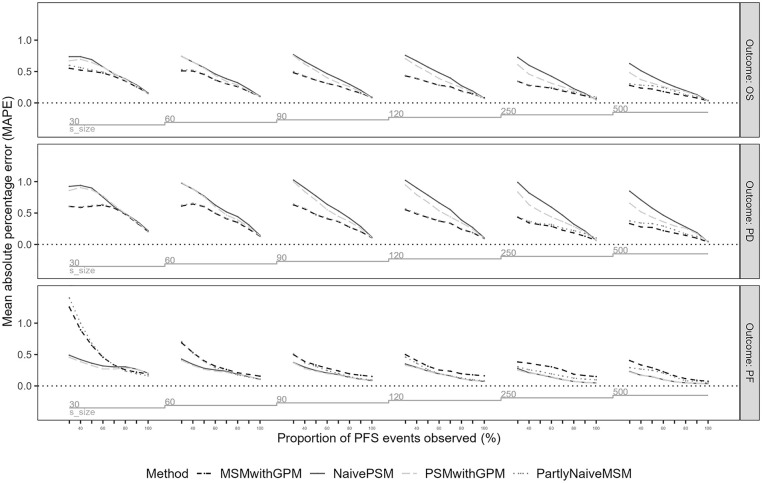
Mean absolute percentage error (MAPE) for mean survival in progression-free (PF), progressed disease (PD), and overall survival (OS) (PF + PD) in the first scenario across model analysis approaches, sample sizes, and follow-up completeness in terms of proportion of events observed.

We found that across all scenarios, smaller samples and more limited follow-up were associated with larger error (MAPE) (Figure 3, Appendix Figure 6) for both methods. With complete follow-up, both PSM and MSM model types were able to accurately capture the population mean survival, with the caveat that there were some additional replications in which MSM estimation failed. If a model could be estimated, MSM generated similar or more accurate mean survival than PSM approaches did. With short follow-up, mean OS was still subject to considerable absolute error of several life-years in magnitude across each scenario.

The largest errors were observed for datasets with limited follow-up when using PSM. Naïve PSM produced larger errors with limited follow-up than PSM with GPM in all scenarios except 2, in which event hazards were increasing over time. Larger absolute errors were observed for naïve PSM in scenarios 3 and 4, when a longer extrapolation time horizon was required (Appendix Figures 6 and 7). In percentage error, the performance of PSM was worst for scenario 3, when disease-related mortality risks were decreasing in the observed trial data, with a mean percentage error above 100% difference from the true mean with limited follow-up and considerable error remaining for small samples even with full follow-up.

Results of partly naïve MSM were not consistently different from that of MSM with GPM in estimating overall life-years (OS) for scenarios 1 to 3. Partly naïve MSM produced larger errors for time spent in PF in scenarios 3 and 4, where event hazards were decreasing. The largest differences were observed for scenario 4, where the error was larger with partly naïve MSM for both PFS and OS.

Across all scenarios for all methods, the magnitudes of error for OS were much larger than that of PF. Errors associated with time spent in PF were more similar across modeling approaches. Time spent in PF demonstrated patterns similar to that seen in previous studies of extrapolation performance for an initial or single event.^10,11^

We also examined mean absolute error in years and coverage (more details are given in Appendix Figures 7 and 8); interpretations were consistent with those of MAPE.

## Discussion

This analysis provided important insights into the performance of different modeling approaches used for economic evaluations that capture time dependency with limited clinical data. Both MSM and PSM model types were able to accurately capture the mean survival of the populations when there was complete or near-complete follow-up. Previous studies have considered error for mean survival in a single outcome, and our results are consistent with the findings of larger error when data are more limited due to small samples or limited follow-up leading to a large proportion of censored data.^10,11^ However, we found important differences in absolute error for estimated mean survival with multiple events using the different model types. When fitting parametric distributions to the data using MSM, with very small samples it was slightly more likely that a hazard would fail to be fit among all 6 distributions used for at least 1 of the transitions or events modeled. This is important because the inability to estimate at least 1 of the hazards means that an economic evaluation cannot be created with the data. When a model could be estimated for each event hazard, results with both model types were subject to considerable error with short follow-up. However, across scenarios, MSM produced similar or smaller error in mean survival than PSM approaches did, particularly PSM that did not incorporate baseline GPM hazard. Very large errors were observed using PSM with limited follow-up for the cohort; in some cases, with more than 100% error from the true population mean OS. This magnitude of error can translate into multiple life-years across our scenarios, which would have a considerable impact in a decision model–based economic evaluation. This finding provides empirical evidence for the risk of considerable error in results when extrapolating to a lifetime horizon with naïve PSM using limited data.^7^ Across MSM and PSM methods, the magnitudes of error for time spent in OS were much larger than that of PF, indicating risk of extrapolation error when using limited clinical data could be exacerbated with additional distant clinical endpoints.

Mortality is often assumed to be driven by the disease when studying therapies, especially in an aggressive illness like many advanced cancers. As a result, general mortality from other causes is considered unlikely to be the reason for death for most patients during a clinical trial. Thus, when extrapolating, an analyst might assume that the disease-related hazard will dwarf GPM hazards and ignore its effects. However, we found that regardless of the pattern of disease-specific mortality risks, inclusion of GPM mortality trends when extrapolating to lifetime horizons aid in the plausible capture of overall population survival. Different approaches for incorporating baseline GPM hazards into parametric survival curves have been highlighted in the literature.^14,23^ Our study was consistent with previous studies reporting that GPM hazards improve parametric extrapolation,^23^ although some of the effects were modest. The importance of this approach has not been examined when incorporating parametric distributions into economic decision models, particularly in the context of different decision-modeling methods. Analysts may incorporate GPM into PSM models using more simplistic methods, such as converging hazards, whereby the larger of the GPM or cause-specific hazard at each time point is selected. We estimated hazards using internal additive hazards as most clinically appropriate and recommended in previous research,^23^ functionality that is easily applied in existing survival analysis packages in R (*flexsurv*),^22^ and incorporated the resulting survival distribution parameters along with GPM hazards into PSM or MSM model structures. There are other potential approaches to extrapolating clinical trial data in a decision model with multiple health states within PSM or MSM frameworks worthy of future research to identify best practices.

Despite the benefits from explicitly modeling the causal relationships with an MSM framework, a commonly cited concern is that clinical trial data are often insufficient for estimating all event hazards for MSM.^7^ This issue was observed in our analysis, in that at least 1 of the event hazards failed to be estimated in a larger proportion of replications than for PSM, particularly when small samples or limited follow-up meant there were few or no events from which to estimate hazards for PD to death. Although we directly estimated length of stay with DES for computational efficiency in this simulation, we could have extrapolated using discrete-time MSM-based microsimulation with the same fitted hazards or a semi-Markov cohort model employing tunnel states to capture time dependency, both of which are more computationally demanding. Regardless, the underlying data fitting would result in the same challenges with respect to the need for sufficient data to fit parametric models or need for simplifying assumptions or external data. It is important to recognize that supplementation with external data sources is commonplace in health state transition-based modeling methods when downstream data are not available or sufficient from the primary clinical data, but care must be taken given the supplemented hazards may not reasonably replicate the disease process even if the structure is appropriate.

There are several limitations of this analysis. We attempted to incorporate heterogeneity through a range of potential baseline ages and differences between GPM data and distributions. We applied different mortality laws in the data-generation process and in extrapolation, but we recognize that the distributions produce similar estimates, possibly contributing to the accuracy of the simulation results; however, as accurate results were observed for only some of methods examined, the findings must at least in part be caused by differences in methods and not simulation design alone. Further, we purposely misspecified GPM in the population data-generation process by using mortality data from 4 decades earlier. The difference did not negatively affect the overall estimation incorporating the appropriate trend in increasing mortality with age. In addition, on the analysis side, for GPM extrapolation we fit life table data at 1 fixed starting age of 60 y for all individuals, as a simplification that could be easily applied by a researcher even without access to individual patient data. These simplifications also did not obscure the benefit derived from incorporating GPM hazards for a more heterogenous population. However, more refined data could be obtained and applied for a trial-specific cohort with age- and country-specific hazards for each patient to further improve estimation. Regardless, these findings are reassuring that capturing the general shape of GPM hazards benefits estimation without exact precision about the magnitude. In an era in which global economic models are built from multinational trials and adapted to an individual country’s HTA process with local data, further work is needed to determine the best practice for characterizing GPM and applying life table data to inform estimation within individual countries from global studies.

Our population data were idealized in that a patient whose disease worsens very rapidly would possibly be clinically observed as dying from the PF state, while a rapid occurrence of both events in sequence is “observed” in our data; although true to the reality of intermittent observations in clinical trials, this limitation could be further examined in future studies. In addition, common diseases with a larger contribution to general mortality (e.g., cardiovascular diseases) might require further refinement to define hazards for death due to other causes from GPM data. In the analysis, we simplified the parametric distribution fitting process relative to best practice and relied solely on information criteria to select best-fitting curves, ignoring other assessments for plausibility or appropriateness.^27,28^ We used the simplest estimation approach for naïve PSM, ignoring even crude GPM approaches such as converging OS hazards, which retains the larger of the extrapolated or GPM hazards at any given time.^29^ We did not include discounting for effects, which would reduce the overall magnitude of life-years and thus would likely also reduce the magnitude of error, but this would be unlikely to change the implications of the comparisons of relative error between methods. Similarly, there are also different approaches to fitting DES models, and although some differences may exist between methods,^30^ differences would likely be small, and the method selection would not be consequential in comparison with PSM. While most files are unlikely to have sample sizes as small as the lowest group we tested, there are oncology drugs considered for funding based on very small sample sizes from early-phase evidence, subgroups, or basket trials. Of 25 oncology submissions to HTA in Canada in 2023, 8 were based on early-phase data, and all but 1 of the pivotal trials involved fewer than 100 patients; recent reviews for tumor-agnostic (NTRK-targeted) therapies included trials of 58 to 118 patients, and the assessment of subgroups was conducted by tumor site with fewer than 30 patients each to inform economic modeling.^31,32^ The challenges in these scenarios for estimation and the exploration of alternative approaches indicate the relevance of our findings to these settings.^33^ Finally, we examined only single-arm results and did not explore the implications for comparative analysis between 2 treatments, as would be essential to the estimation and interpretation in practice for HTA. Commonly, a treatment parameter is included in parametric survival models to capture and extrapolate relative treatment effects from observed data. It is unknown whether the implications of this study will hold when including relative treatment effects in extrapolation to estimate incremental survival benefits. Further studies will be needed to confirm the generalizability across a wider range of scenarios and the relevance of these findings in comparative settings.

In conclusion, we found that when extrapolating clinical trial data with multiple endpoints, caution should be taken with all modeling approaches when underlying data are very limited due to the potential for the large magnitude of error. PSM approaches with limited follow-up can lead to large magnitude errors. Incorporating baseline GPM hazards slightly improves the accuracy of total life-years, but with limited data, this approach does not meaningfully improve error, and the estimation of time spent in individual health states not affected by GPM adjustment (i.e., PF in our study) is still subject to error, leading to misattribution of survival partitioning between states, which has implications for appropriately capturing costs and utilities in an economic model. MSM approaches led to more convergence failures due to limited data, but when estimable, our simulation showed that MSM models incorporating time-dependent hazards are expected to perform similarly to or better than PSM in estimating the mean survival with limited data. Further studies are needed to address challenges with the estimation of MSM and similar state transition-based modeling methods with limited data and assess the applicability of these findings to comparative analyses estimating incremental survival benefits.

## Supplemental Material

sj-docx-1-mdm-10.1177_0272989X251342596 – Supplemental material for Impact of Limited Sample Size and Follow-up on Partitioned Survival and Multistate Modeling-Based Health Economic Models: A Simulation Study Supplemental material, sj-docx-1-mdm-10.1177_0272989X251342596 for Impact of Limited Sample Size and Follow-up on Partitioned Survival and Multistate Modeling-Based Health Economic Models: A Simulation Study by Jaclyn M. Beca, Kelvin K. W. Chan, David M. J. Naimark and Petros Pechlivanoglou in Medical Decision Making
