# Supplementary material for: Impact of Limited Sample Size and Follow-up on Partitioned Survival and Multistate Modeling-Based Health Economic Models: A Simulation Study
Source: Med Decis Making. 2025 Jun 25;45(6):714–25. doi: 10.1177/0272989X251342596 (PMC12260197; doi:10.1177/0272989X251342596)
Supplement: sj-docx-1-mdm-10.1177_0272989X251342596 – Supplemental material for Impact of Limited Sample Size and Follow-up on Partitioned Survival and Multistate Modeling-Based Health Economic Models: A Simulation Study [file sj-docx-1-mdm-10.1177_0272989X251342596.docx]

## Appendix

### More details about population data generation

The data for each population was generated from a multistate process depicted in Appendix Figure 1, using hazards (or transition intensities) described in the following data generating transition intensity matrix (TIM) for transitions between PF, PD, Death from other causes, Death from disease. Transition intensities represent the instantaneous risk of moving from one state to another in a multistate process as a function of time.

$$TIM=\left[ \begin{matrix} -\lambda_{prog}\left( t \right)-\lambda_{gpm}(t) & \lambda_{prog}(t) & \lambda_{gpm}(t) & 0 \\ 0 & -\lambda_{gpm}\left( t \right)-\lambda_{dsm}(t) & \lambda_{gpm}(t) & \lambda_{dsm}(t) \\ 0 & 0 & 0 & 0 \end{matrix} \right]$$

**Appendix Figure 1.** Model diagram with staggered entry from pre-trial state into progressive disease model for simulating hypothetical population data


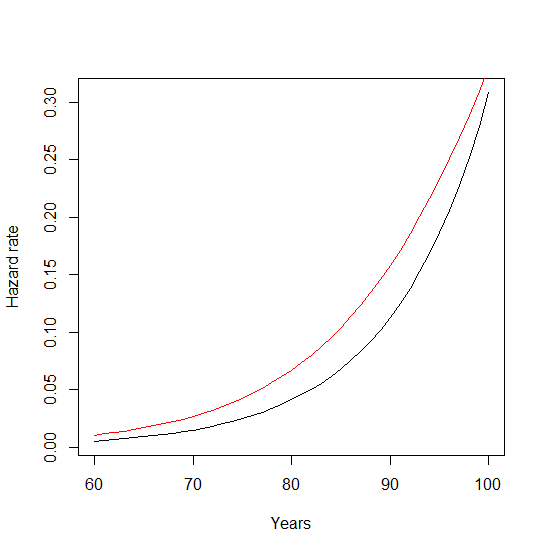


**Appendix Figure 2. Difference in GPM hazard used in data generating process (red curve) and for analysis (black curve)**


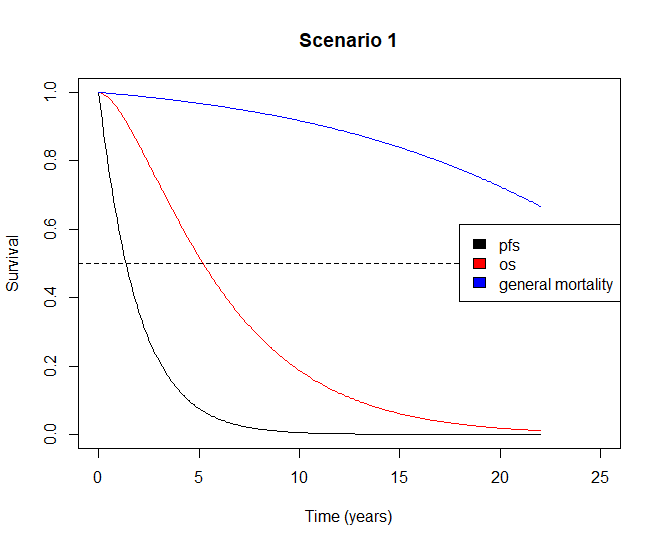

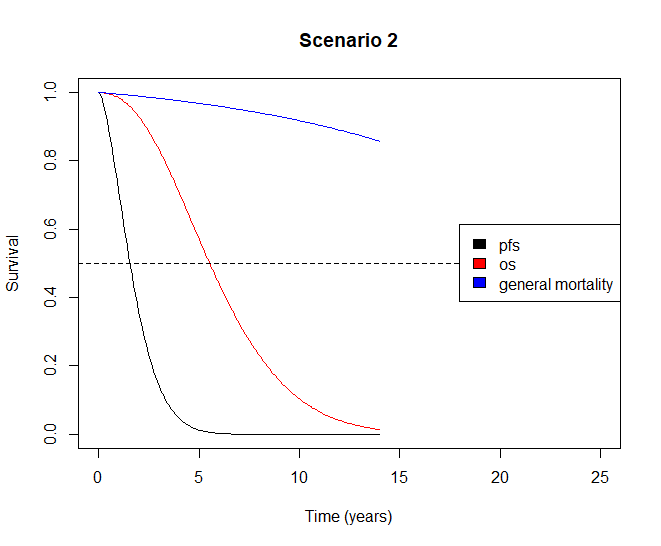

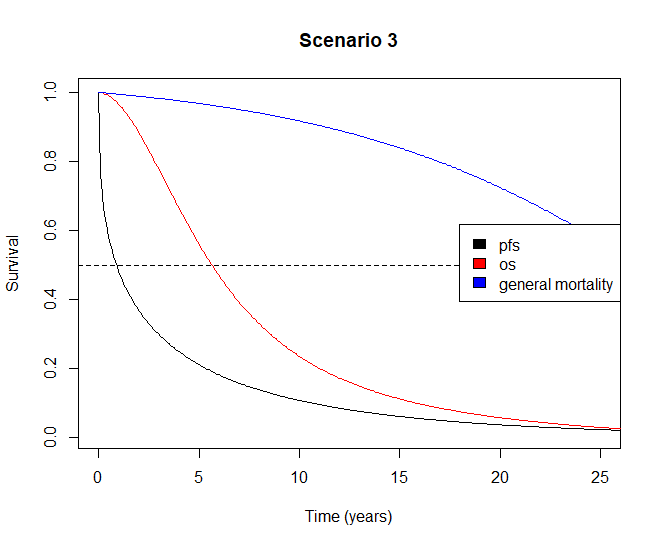

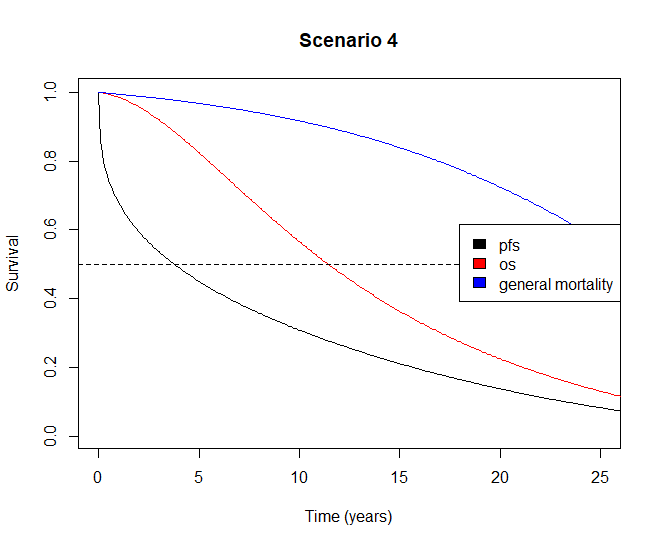


**Appendix Figure 3. Scenario population survival curves**


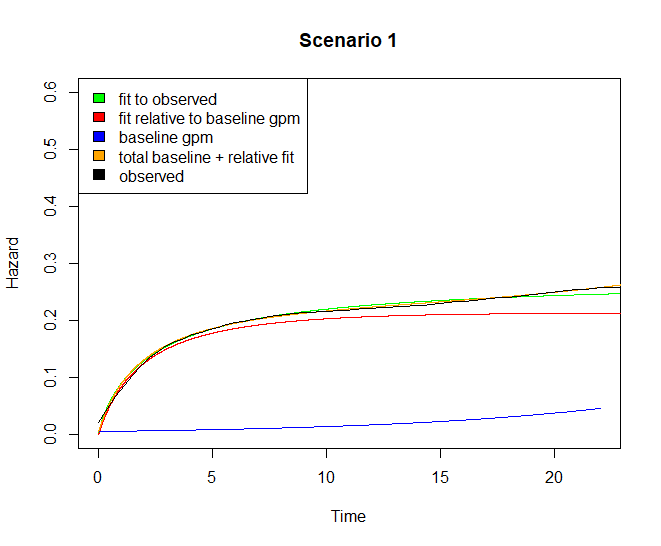

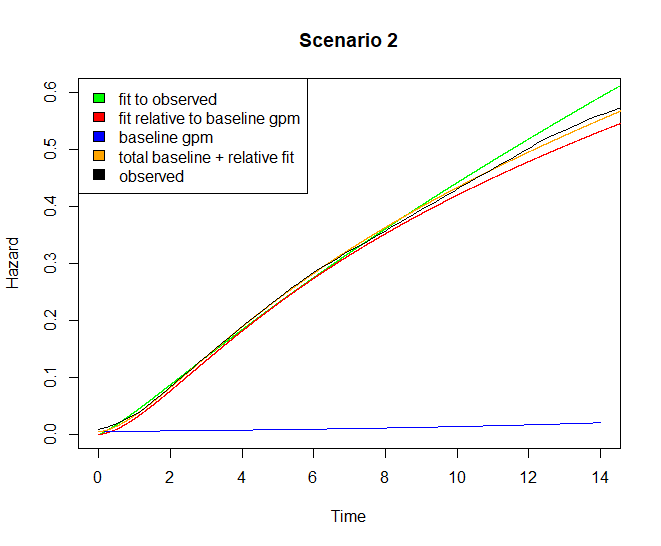

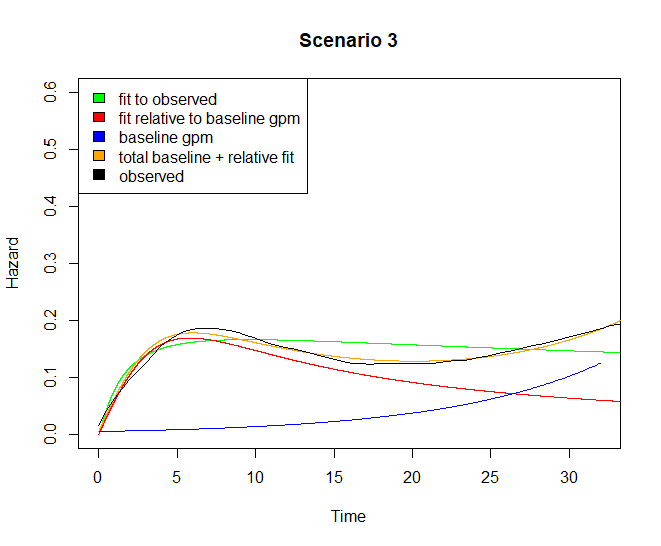

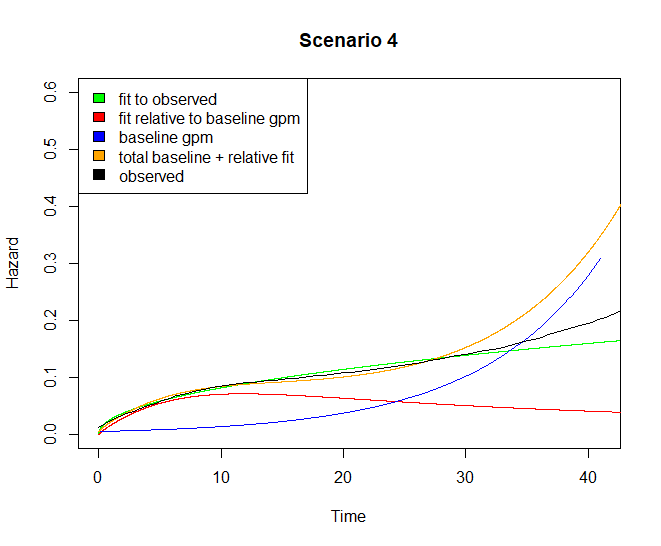


**Appendix Figure 4. Scenario population hazard curves**

Scenario 1: Constant hazards (exponential) for progression and for excess mortality from PD

Scenario 2: Monotonically increasing hazards (Weibull) for progression and for excess mortality from PD

Scenario 3: Monotonically decreasing hazards (Weibull) for progression and monotonically increasing hazards (Weibull) for excess mortality from PD

Scenario 4: Monotonically decreasing hazards (Weibull) for progression and monotonically increasing hazards (Weibull) for excess mortality from PD, similar to scenario 3 with lower rates

In all scenarios, Kannisto-Makeham mortality law fitted to life-tables from Statistics Canada from a historical baseline year (1980/82) was used for general population mortality (GPM) from PFS and from PD

**
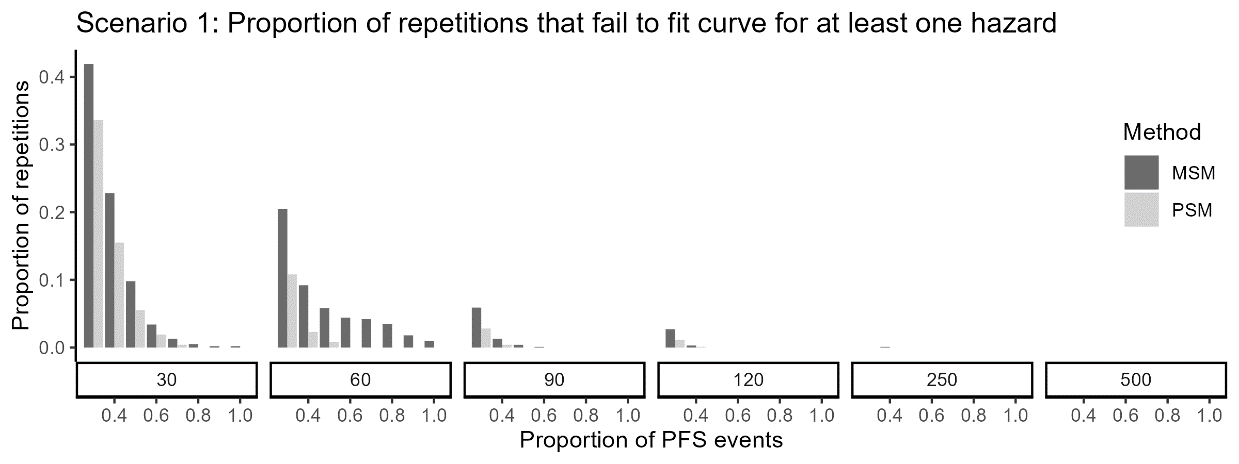
** **
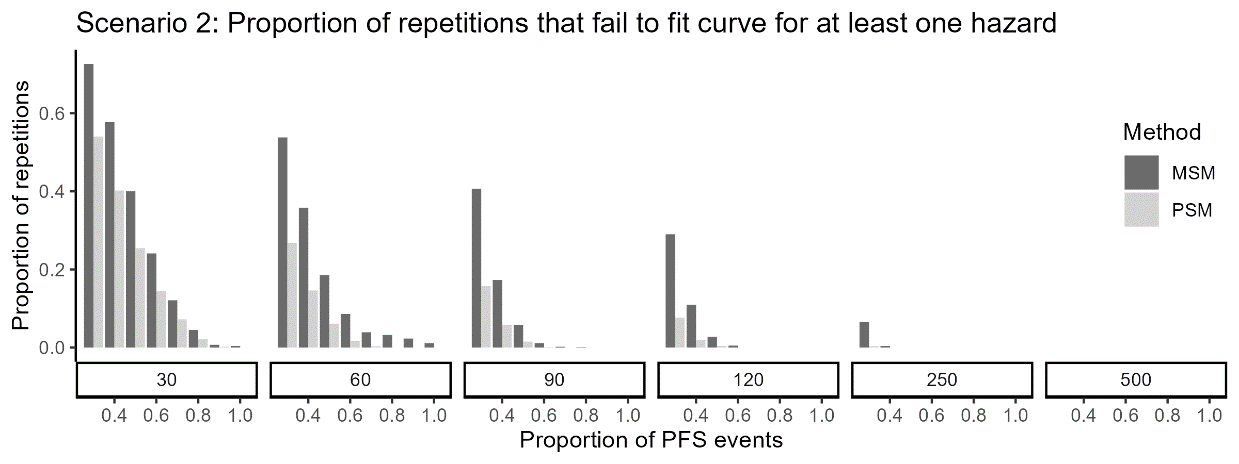

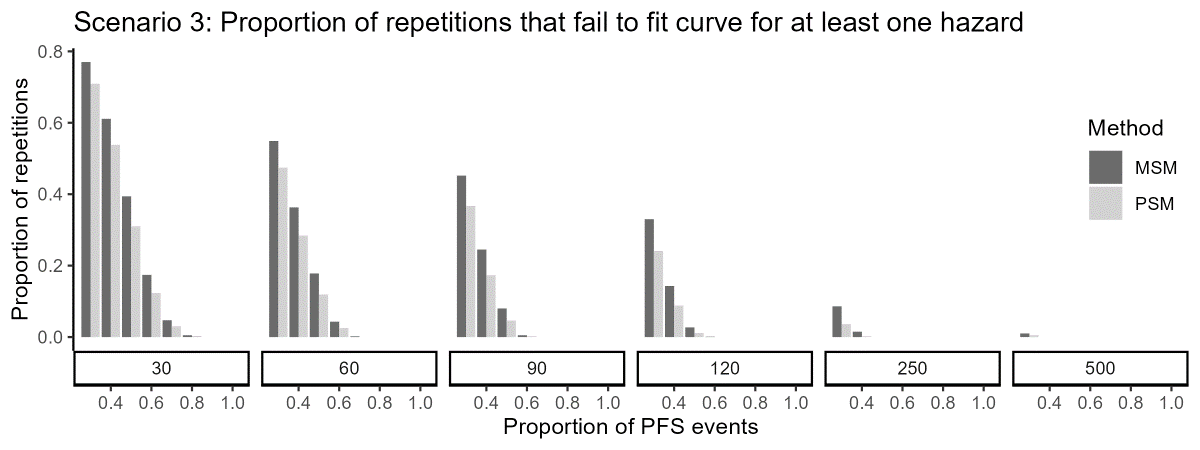
**


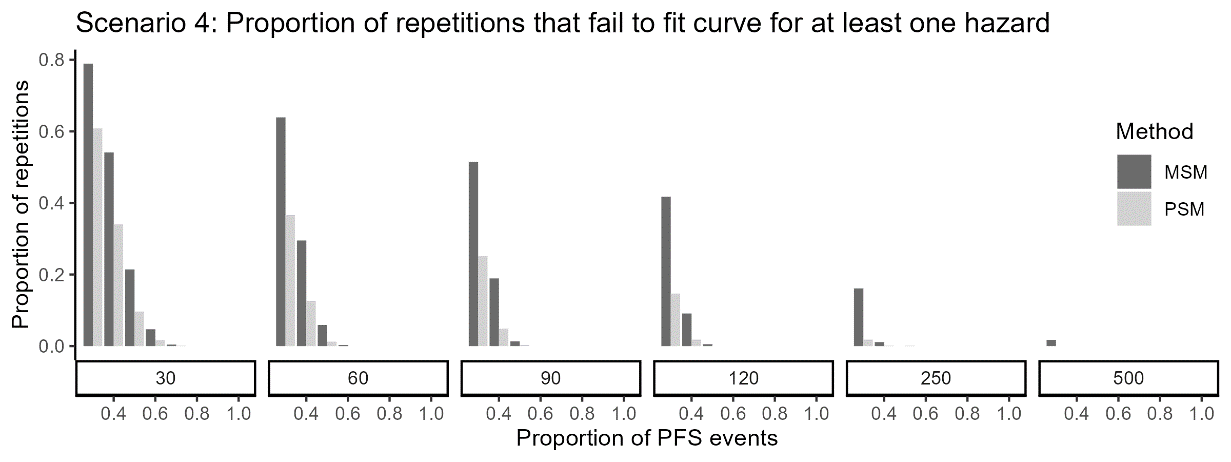


**Appendix Figure 5. Proportion of replications where estimation of at least one hazard (for all six distributions tested) fails to converge across scenarios 1-4**

### More details about performance

Mean absolute percentage error and mean absolute error across scenarios are shown in Appendix Figures 6 and 7. There is some variability across scenarios especially at very low sample sizes, but the overall pattern is similar in that the error is reduced when follow-up is more complete, and the performance of PSM is frequently worse than MSM approaches.

Coverage was strongly associated with sample size and follow-up and was very poor for naïve PSM unless follow-up was nearly complete (Appendix Figure 8). Partly naïve MSM demonstrated worsening coverage with longer-follow-up. Coverage with MSM with GPM was only reliably close to nominal estimates with near-complete follow-up.

**
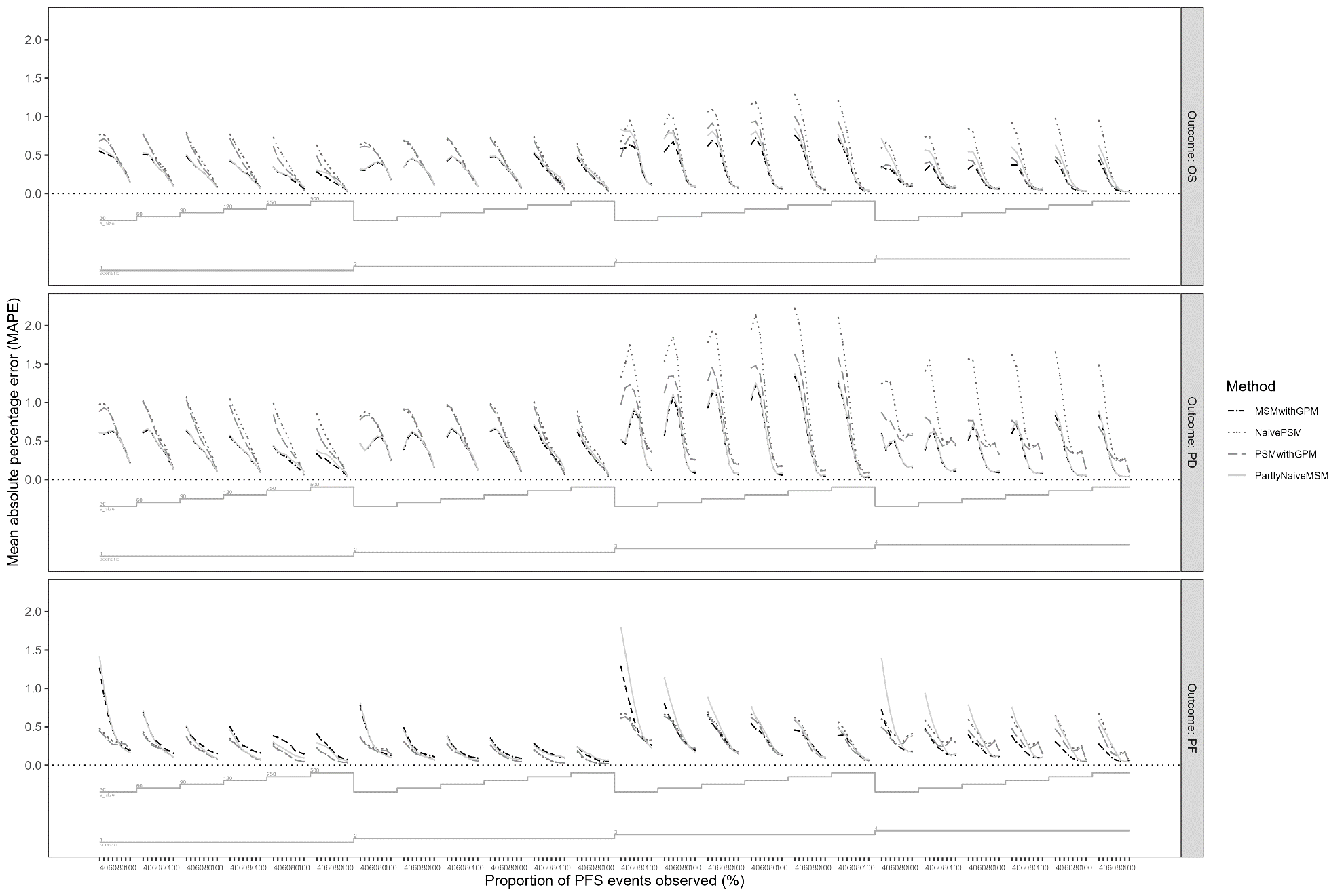
**

**Appendix Figure 6. Mean Absolute Percentage Error (MAPE) across scenarios, varying sample size and events observed**

**
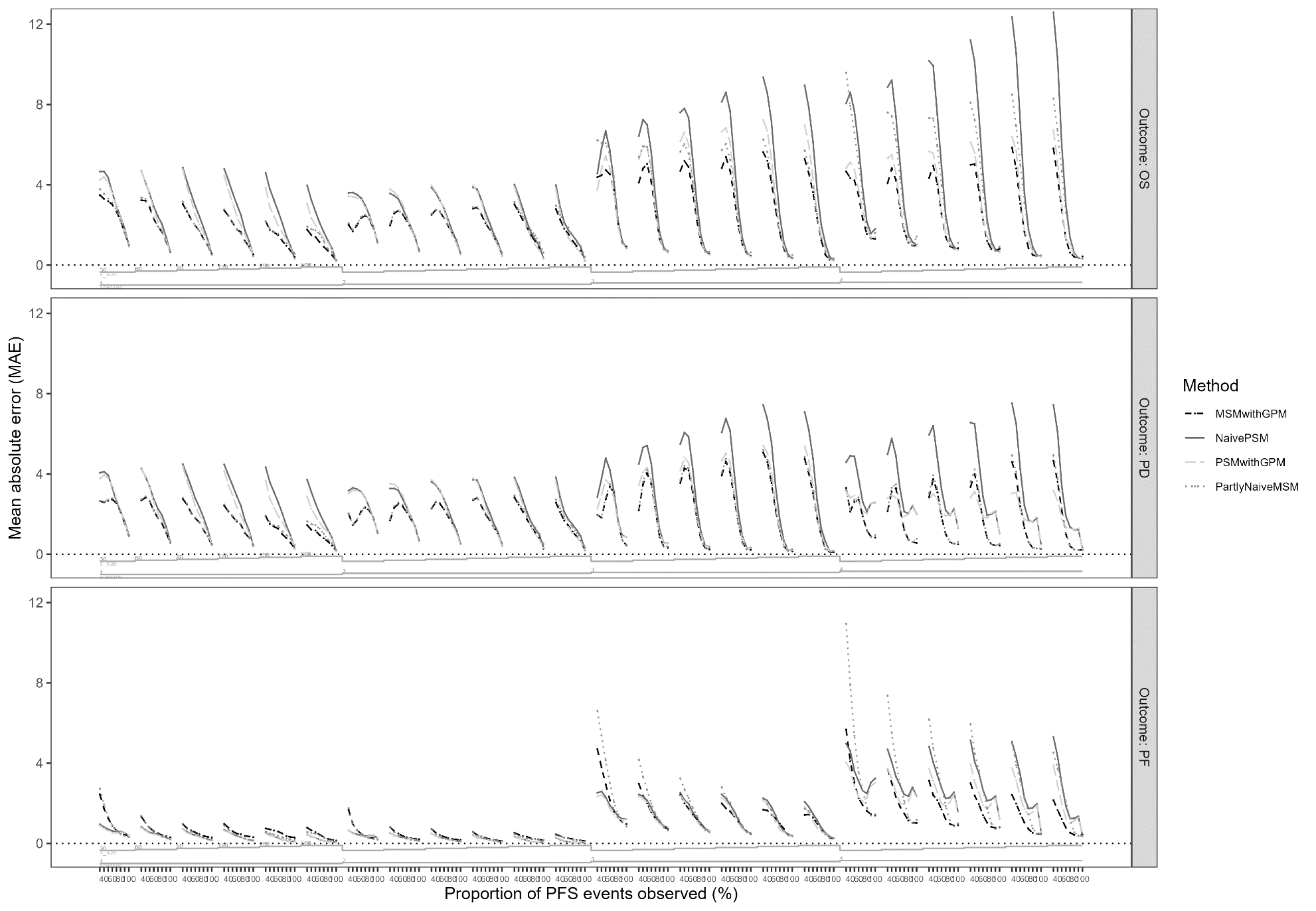
**

**Appendix Figure 7. Mean Absolute Error (MAE) in life years across scenarios, varying sample size and events observed**

**
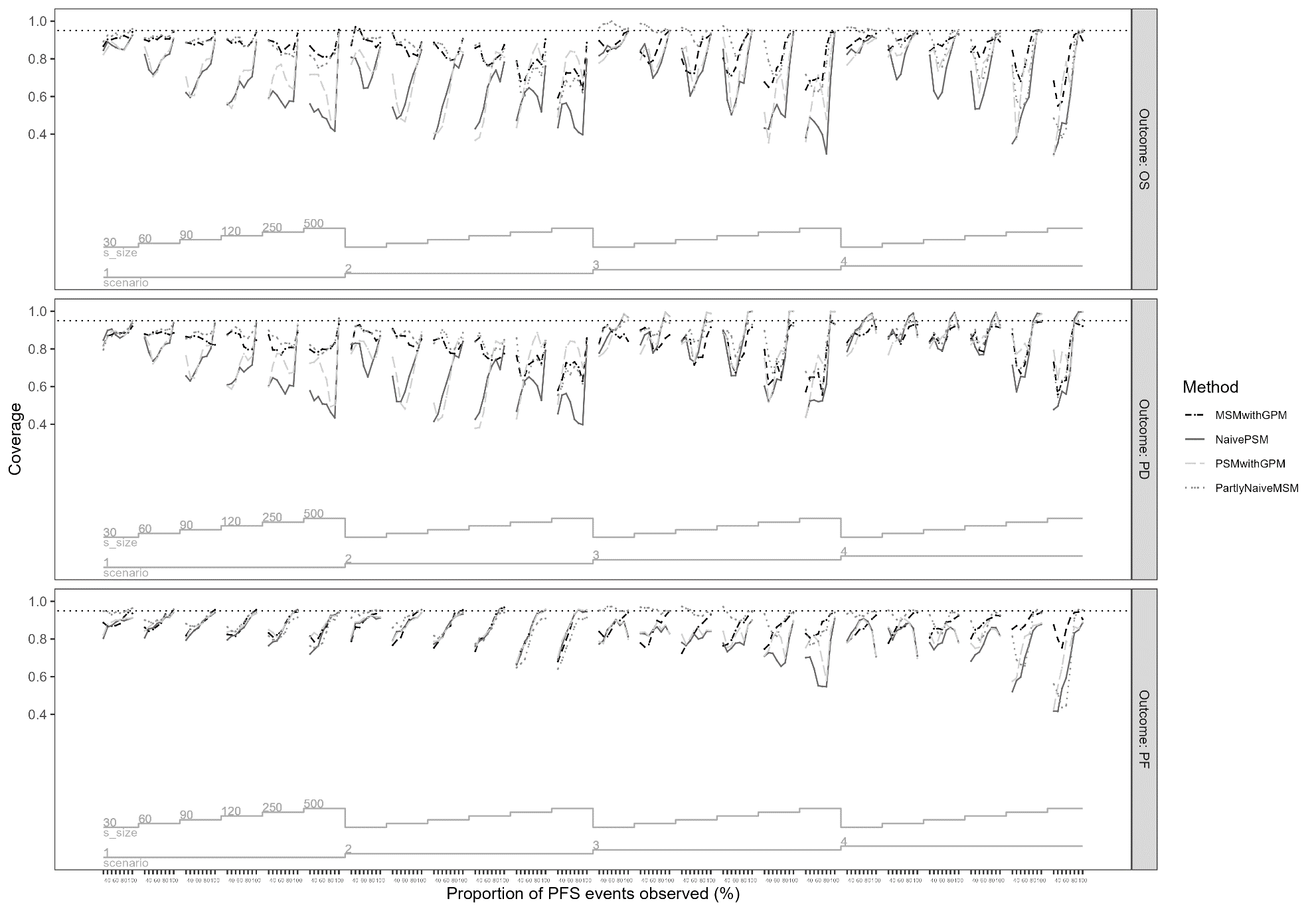
**

**Appendix Figure 8. Coverage across scenarios, varying sample size and events observed**
